# Antimicrobial resistance is widespread among intestinal and extra-intestinal *Bacteroides fragilis* strains

**DOI:** 10.1128/iai.00529-25

**Published:** 2025-11-24

**Authors:** Marvic Carrillo Terrazas, Renee E. Oles, Luke R. Loomis, Chia-Yun Hsu, Adriana Vasquez Ayala, Michael H. Lee, Mousumi Paulchakrabarti, David Pride, Biswa Choudhury, Victor Nizet, Manuela Raffatellu, Rob Knight, Hiutung Chu

**Affiliations:** 1 Department of Pathology, University of California, San Diego 189207, La Jolla, California, USA; 2 Division of Host-Microbe Systems and Therapeutics, Department of Pediatrics, University of California, San Diego 547075, La Jolla, California, USA; 3 GlycoAnalytics Core, University of California San Diego 8784, La Jolla, California, USA; 4 Center for Microbiome Innovation, University of California, San Diego 8784, La Jolla, California, USA; 5 Center for Innovative Phage Applications and Therapeutics (IPATH), University of California, San Diego 8784, La Jolla, California, USA; 6 Center of Advanced Laboratory Medicine (CALM), University of California, San Diego 8784, La Jolla, California, USA; 7 Department of Pediatrics, School of Medicine, UC San Diego 547075, La Jolla, California, USA; 8 Skaggs School of Pharmacy and Pharmaceutical Sciences, UC San Diego 15500, La Jolla, California, USA; 9 Chiba University-UC San Diego Center for Mucosal Immunology, Allergy and Vaccines (cMAV), University of California, San Diego 8784, La Jolla, California, USA; 10 Shu Chien-Gene Lay Department of Bioengineering, University of California, San Diego 207027, La Jolla, California, USA; 11 Department of Computer Science and Engineering, University of California, San Diego 214553, La Jolla, California, USA; 12 Halıcıoğlu Data Science Institute, University of California, San Diego 684628, La Jolla, California, USA; University of California, Davis, Davis, California, USA

**Keywords:** *Bacteroides fragilis*, antimicrobial resistance, genome-wide association study

## Abstract

*Bacteroides fragilis* is an important member of the human gut microbiota, where it contributes to immune modulation, intestinal barrier integrity, and colonization resistance. Despite its beneficial roles as a symbiont in the gut, *B. fragilis* is also the most commonly isolated anaerobe in clinical infections, implicated in intra-abdominal abscesses, bloodstream infections, and soft tissue infections. Antimicrobial resistance (AMR) is increasingly recognized as a major factor in its transition from symbiont to opportunist; however, the relationship between resistance and anatomical site of isolation remains poorly defined. Here, we compared AMR phenotypes and genotypes between intestinal and extra-intestinal *B. fragilis* isolates to assess whether clinical strains are enriched for resistance determinants. Surprisingly, we found comparable susceptibility profiles and AMR gene content between the two groups. Minimal inhibitory concentrations (MICs) were broadly similar, and β-lactamase activity was detected in ~70% of the isolates regardless of the isolation site. We found that resistance genes were similarly distributed across both intestinal and clinical strains. A microbial genome-wide association study (mGWAS) confirmed the known resistance markers, such as *ermF*, *aadS*, and *tetQ,* and identified novel associations with conjugative transposons, efflux transporters, regulatory genes, and previously uncharacterized loci. These findings suggest that intestinal strains serve as a reservoir of clinically relevant resistance determinants that may be mobilized under selective pressure. Although prior work has largely focused on clinical isolates, our findings highlight the need to surveil AMR within the gut microbiota, where widespread resistance in commensal bacteria has the potential to complicate treatment of extra-intestinal infections.

## INTRODUCTION

*Bacteroides fragilis* is a prominent member of the human gut microbiota, where it contributes to immune modulation, intestinal barrier integrity, and colonization resistance (1–3). Despite its beneficial role as a gut commensal, *B. fragilis* is also the most frequently isolated anaerobe in clinical infections, including intra-abdominal abscesses, bloodstream infections, and soft tissue infections (4). This duality raises fundamental questions about the mechanisms that drive *B. fragilis* from a gut symbiont to a pathobiont (5). One critical factor facilitating *B. fragilis* pathogenicity is antimicrobial resistance (AMR), which complicates treatment strategies and contributes to persistence during extra-intestinal infections. Current classification and recognition of resistance phenotypes within *B. fragilis* have segregated the species based on the presence of antimicrobial resistance genes into two main groups, Division I and Division II (6). Division I strains are *cepA*-negative and carry a β-lactamase, whereas division II strains are *cfiA* or *ccrA*-positive and encode a metallo-β-lactamase (7). *B. fragilis* is also part of a diverse microbial ecosystem in the human gut, where horizontal gene transfer plays a significant role in shaping bacterial genomes, raising the possibility that commensal strains can serve as reservoirs of resistance determinants. Indeed, AMR genes have been identified in intestinal *B. fragilis* strains (8), highlighting the need to reassess the assumption that antimicrobial resistance phenotypes are primarily relevant during its transient pathogenic state. To further understand the characteristics of the intestinal and extra-intestinal isolates, we investigate AMR phenotypes among *B. fragilis* strains isolated from human sources. Understanding the evolution of AMR in *B. fragilis* provides essential context for interpreting resistance patterns across contemporary intestinal and extra-intestinal isolates.

Initial studies in the 1980s began to track AMR in the species following reports of plasmid-mediated clindamycin resistance (9). At the time, *B. fragilis* was largely susceptible to standard clinical therapies (10), and antimicrobial testing for anaerobes was limited by infrastructure and standardization. In 2004, a national survey conducted for the surveillance of susceptibility patterns among 5,225 *B. fragilis* isolates in the United States revealed that resistance to carbapenems and beta-lactam agents in combination with beta-lactamase inhibitors was found in less than 1% of the total isolates tested; since then, a clear emergence of resistant strains has been observed (9). Despite these trends, the extent to which AMR phenotypes distinguish gut commensals from extra-intestinal strains remains unclear. We recently reported strain-level differences that may contribute to the ecological flexibility of *B. fragilis* (5). Building on this, we now investigate whether AMR phenotypes and resistance gene profiles differ between intestinal and extra-intestinal isolates. Here, we systematically compare AMR phenotypes and resistance gene content between intestinal and extra-intestinal *B. fragilis* isolates from human sources. We also assess long-term trends in AMR gene acquisition by analyzing a historical collection of isolates spanning five decades. Finally, we apply microbial genome-wide association analysis (mGWAS) to identify novel genetic features linked to resistance phenotypes. Together, these efforts aim to clarify whether clinical isolates are uniquely enriched for AMR or whether the gut microbiota already harbors strains with pathogenic potential.

## RESULTS

### Distribution of antimicrobial resistance genes among *B. fragilis*

**Fig 1:**
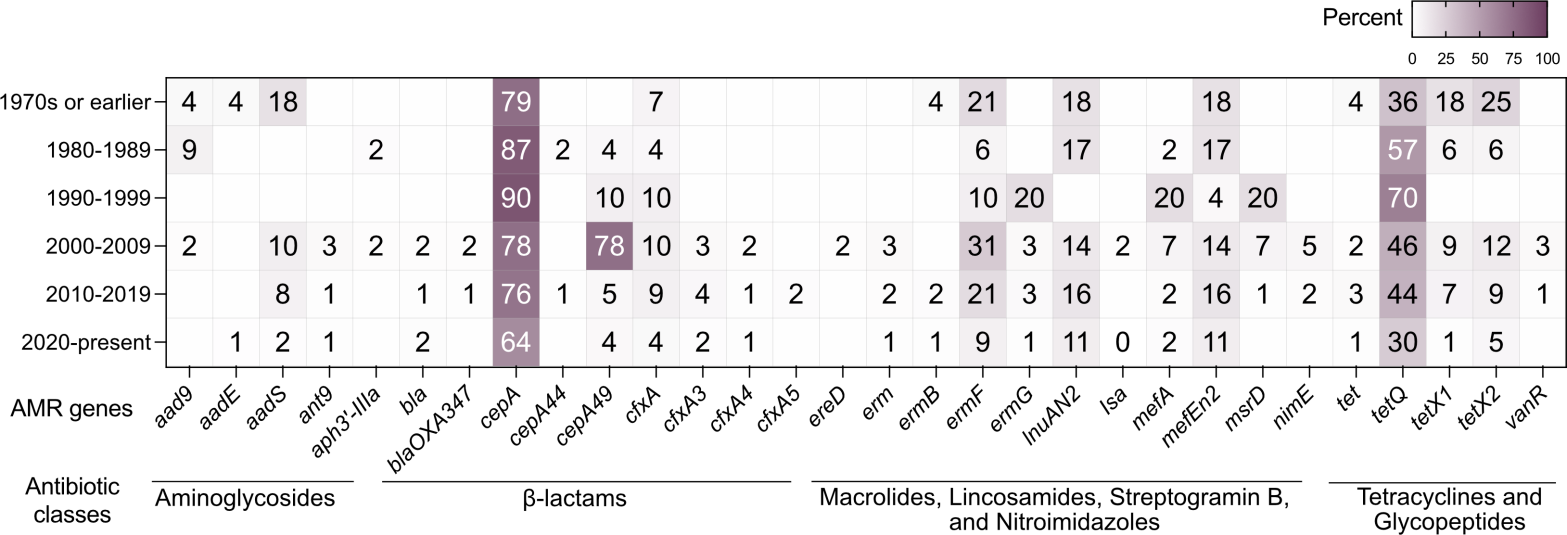
Per decade increase of antimicrobial resistance genes among *B. fragilis* strains. The percentage of strains with the indicated antimicrobial resistance gene, as identified by whole genome sequencing, is shown by decade of isolation: 1970s or earlier (*n* = 6), 1980s (*n* = 37), 1990s (*n* = 9), 2000s (*n* = 28), 2010s, (*n* = 182), and 2020s (*n* = 132). Antimicrobial resistance genes are displayed on the X axis along with antibiotic class, and decade of isolation on the Y axis.

To investigate the long-term trends in AMR, we analyzed *B. fragilis* Division I and II isolates (*n* = 393) spanning from the 1970s to the 2020s. Our data revealed a progressive increase in AMR gene prevalence over time (Fig. 1), particularly between the 1990s and 2000s (Fig. S1). This rise was most notable among genes encoding aminoglycoside resistance (*aadE, aadS*), macrolide resistance (*mefEn2, mefA*), lincosamide resistance (*lnuAN2*), and nitroimidazole resistance (*nimE*). We also observed an increasing prevalence of tetracycline beta-lactamases (*cepA*, *cfx4*, and *bla*) and tetracycline resistance genes (*tetQ, tetX1, and tetX2*), coinciding with the widespread clinical use of cephalosporins and tetracyclines following the "golden age of antibiotics'' in the mid-20th century (11). However, our analysis is limited by the smaller number of strains available from earlier time points, which may introduce sampling bias and limit the resolution of temporal comparisons.These findings are consistent with previous studies that predicted an accumulation of resistance genes in *B. fragilis* due to selective pressure from antibiotic exposure (12, 13), underscoring the impact of antimicrobial use on resistance gene dissemination within the human microbiota.

**Fig 2:**
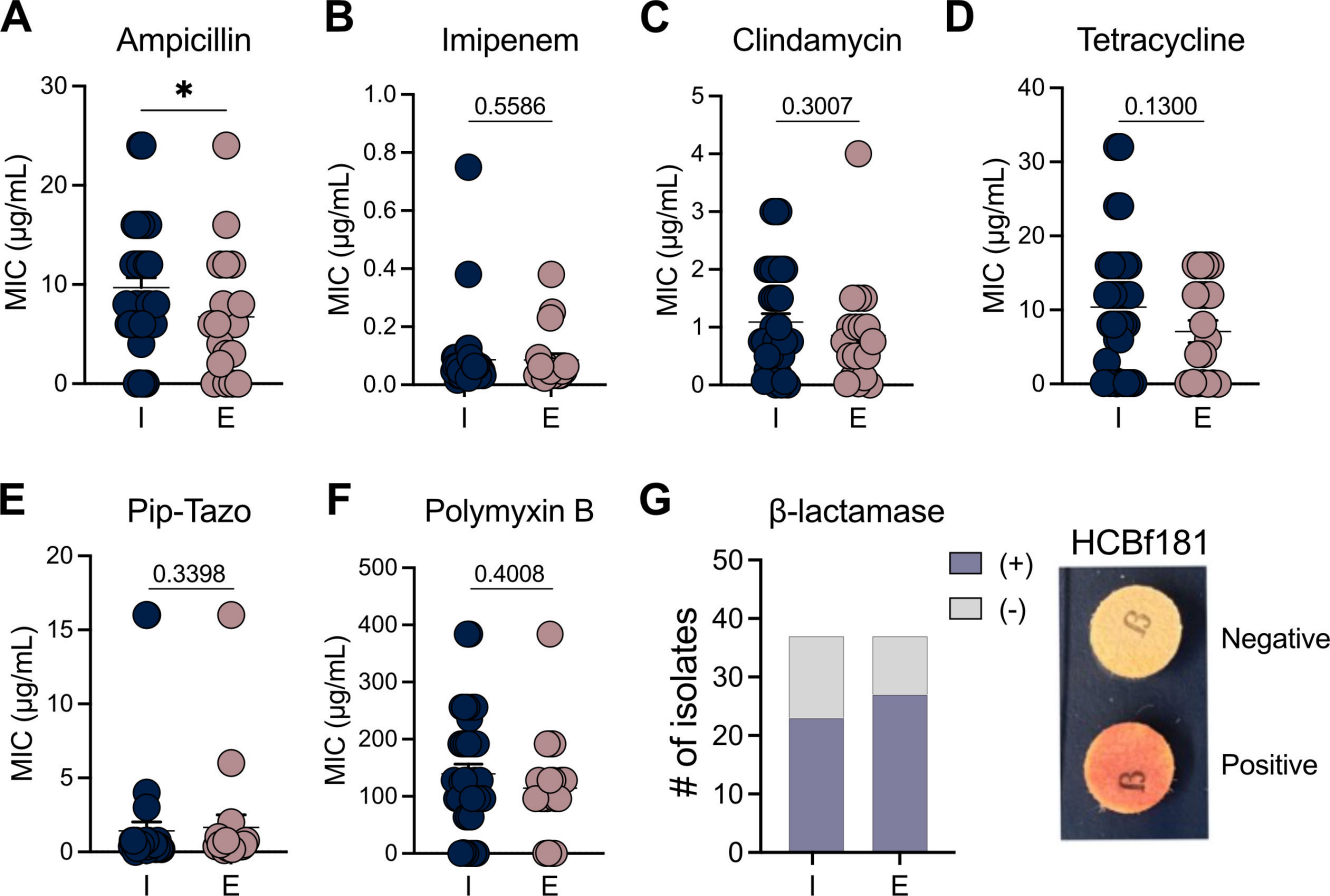
Distribution of antimicrobial resistance among intestinal and extra-intestinal *B. fragilis* strains. (**A–F**) Comparison of antimicrobial minimal inhibitory concentrations (MICs, μg/mL) among intestinal (I) and extra-intestinal (E) *B. fragilis* strains using the Epsilometer test (E test). Antimicrobials tested include: (**A**) ampicillin, (**B**) imipenem, (**C**) clindamycin, (**D**) tetracycline, (**E**) piperacillin-tazobactam (Pip-Tazo), and (**F**) polymyxin B. Unpaired Mann-Whitney test, *P < 0.05. (**G**) β-lactamase production among intestinal (I) and extra-intestinal (E) *B. fragilis* strains measured by chromogenic cephalosporin spot test, Nitrocefin (left) and representative positive reaction for β-lactamase production from strain HCBf181 (right). *n* = 37 per group. *B. fragilis* strains isolated from an unknown source were excluded from the analysis.

Given the observed increase in AMR gene prevalence (Fig. 1), we hypothesized that extra-intestinal isolates may harbor more resistance determinants compared with intestinal strains, reflecting both clinical antibiotic exposure and environmental pressures. To test this, we profiled the antimicrobial susceptibility of 78 *B. fragilis* isolates from both intestinal and extra-intestinal sites, assessing resistance to ampicillin (AMP) (Fig. 2A), imipenem (IMI) (Fig. 2B), clindamycin (CD) (Fig. 2C), tetracycline (TE) (Fig. 2D), piperacillin-tazobactam (TZP) (Fig. 2E), polymyxin B (PB) (Fig. 2F), and beta-lactamase production (Fig. 2G; Table S1). Surprisingly, we observed no significant differences in MIC values between the two groups for most antibiotics tested (Fig. 2B through G), except for ampicillin, where extra-intestinal isolates showed lower MICs than intestinal isolates (*P* = 0.0483, unpaired Mann–Whitney test; Fig. 2A). This result is consistent with the well-documented resistance of members of the *B. fragilis* group (BFG) and *Parabacteroides* spp. to penicillin, with >90% resistance globally (14). Moreover, 70% of *B. fragilis* isolates tested (53 of 78) had exhibited beta-lactamase activity (Fig. 2G), consistent with the known expression of these enzymes in *B. fragilis* (15). Importantly, a few studies have explored resistance profiles among intestinal and extra-intestinal strains, as the Clinical Laboratory Standards Institute (CLSI) guidelines limit antimicrobial susceptibility testing to clinically relevant *B. fragilis* isolates (9, 10). The increasing MIC trend among intestinal strains, compared with extra-intestinal isolates, highlights the role of *B. fragilis* as a commensal microbe that serves as a reservoir of AMR genes that can potentially be transferred to other members of the microbiota during disease states.

### Phylogenetic analysis and AMR gene presence and absence in *B. fragilis* isolates

**Fig 3:**
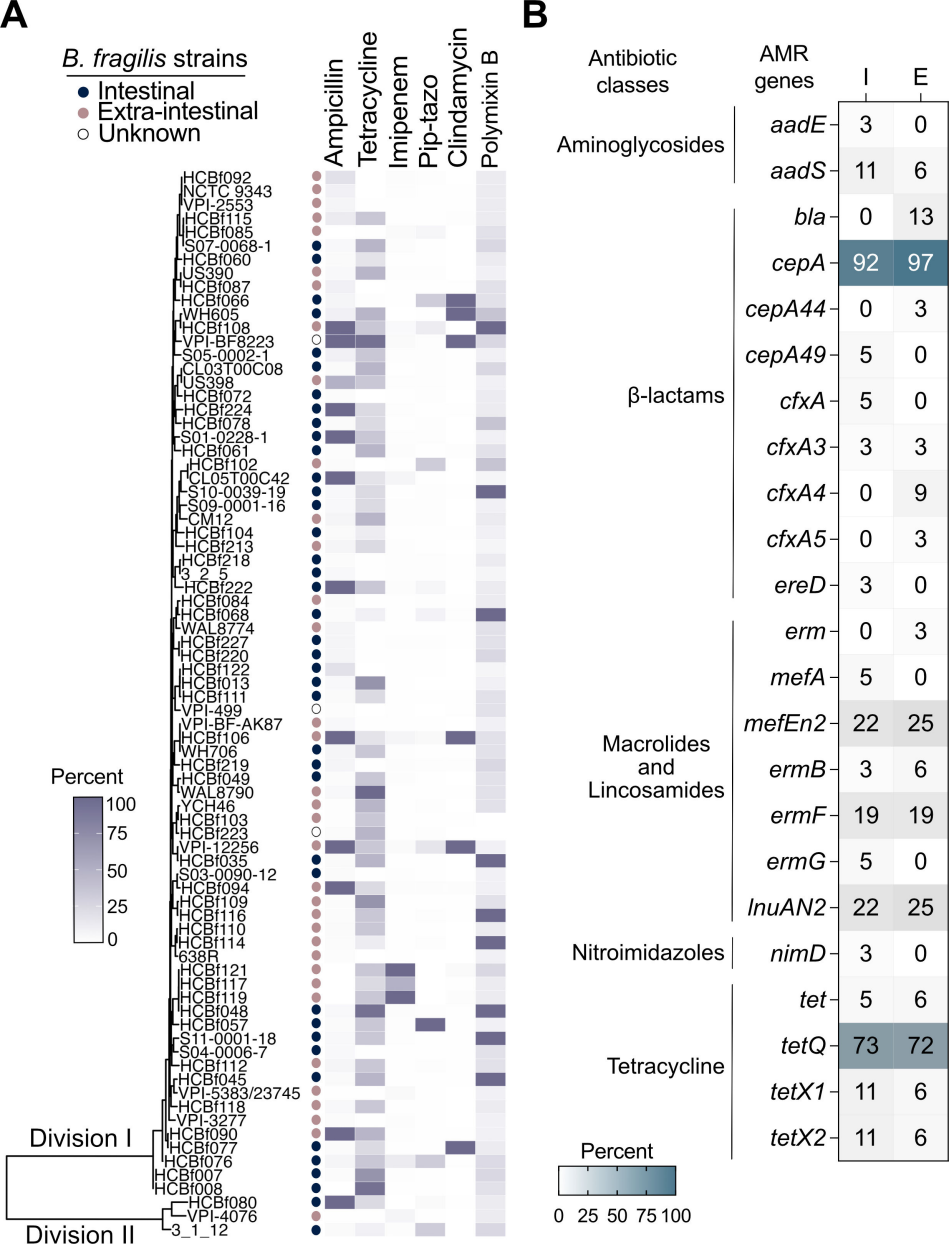
Phylogenetic clustering and AMR profiles of intestinal and extra-intestinal *Bacteroides fragilis* isolates. (**A**) Phylogenetic tree of *B. fragilis* strains, with division II strains present as the outgroup. Tree is annotated with antimicrobial resistance phenotypes (MIC expressed in percentage relative to the max MIC), showing the proportion of resistance to ampicillin, tetracycline, imipenem, piperacillin-tazobactam (pip-tazo), clindamycin and polymyxin B. (**B**) Distribution of antimicrobial resistance genes (AMR) among *B. fragilis* isolated from intestinal (*n* = 37) and extra-intestinal (*n* = 32) sources. Representation in percentage of the presence of AMR genes in intestinal strains and extra-intestinal strains. The average number of AMR genes per strain was 3.13 for intestinal isolates and 3.16 for extra-intestinal isolates.

To further explore the relationship between AMR gene distribution and strain origin, we performed phylogenetic clustering based on the core genome alignment. *B. fragilis* genomospecies are primarily distinguished by their beta-lactamase gene repertoire. Division I strains are typically characterized by the *cepA* gene, the most commonly detected cephalosporinase in this group (8, 16), and by *cfx*, which is more frequently found in clinical isolates with active beta-lactamase activity (17). Of note, these observations are primarily based only on strains isolated outside the gut environment. Therefore, the presence of these determinants in intestinal isolates is likely underrepresented. In contrast, Division II strains are defined by the presence of *cfiA*, a gene encoding a beta-lactamase that confers resistance to carbapenems and beta-lactamase inhibitors (6, 18, 19). We found that intestinal and extra-intestinal isolates did not form distinct phylogenetic clusters, a pattern consistent with our MIC data (Fig. 3A). Instead, the primary separation observed was between Division I and II *B. fragilis* isolates, reflecting previously established genomic distinctions within the species (6, 7, 17, 20). Moreover, the distribution of AMR gene profiles was relatively similar between intestinal and extra-intestinal strains (Fig. 3B; Fig. S2). These findings indicate that both intestinal and extra-intestinal *B. fragilis* strains harbor comparable resistance gene profiles and antimicrobial susceptibility patterns, further supporting that intestinal strains may serve as reservoirs of AMR determinants (16). Contrary to our initial hypothesis, AMR gene enrichment is not exclusive to clinical isolates, underscoring the idea that *B. fragilis* pathogenic potential is highly context-dependent, influenced by the host immune response, niche (e.g., intestinal and extra-intestinal), microbial community dynamics, and potential horizontal gene transfer under selective pressures (e.g., antibiotic exposure).

**Fig 4:**
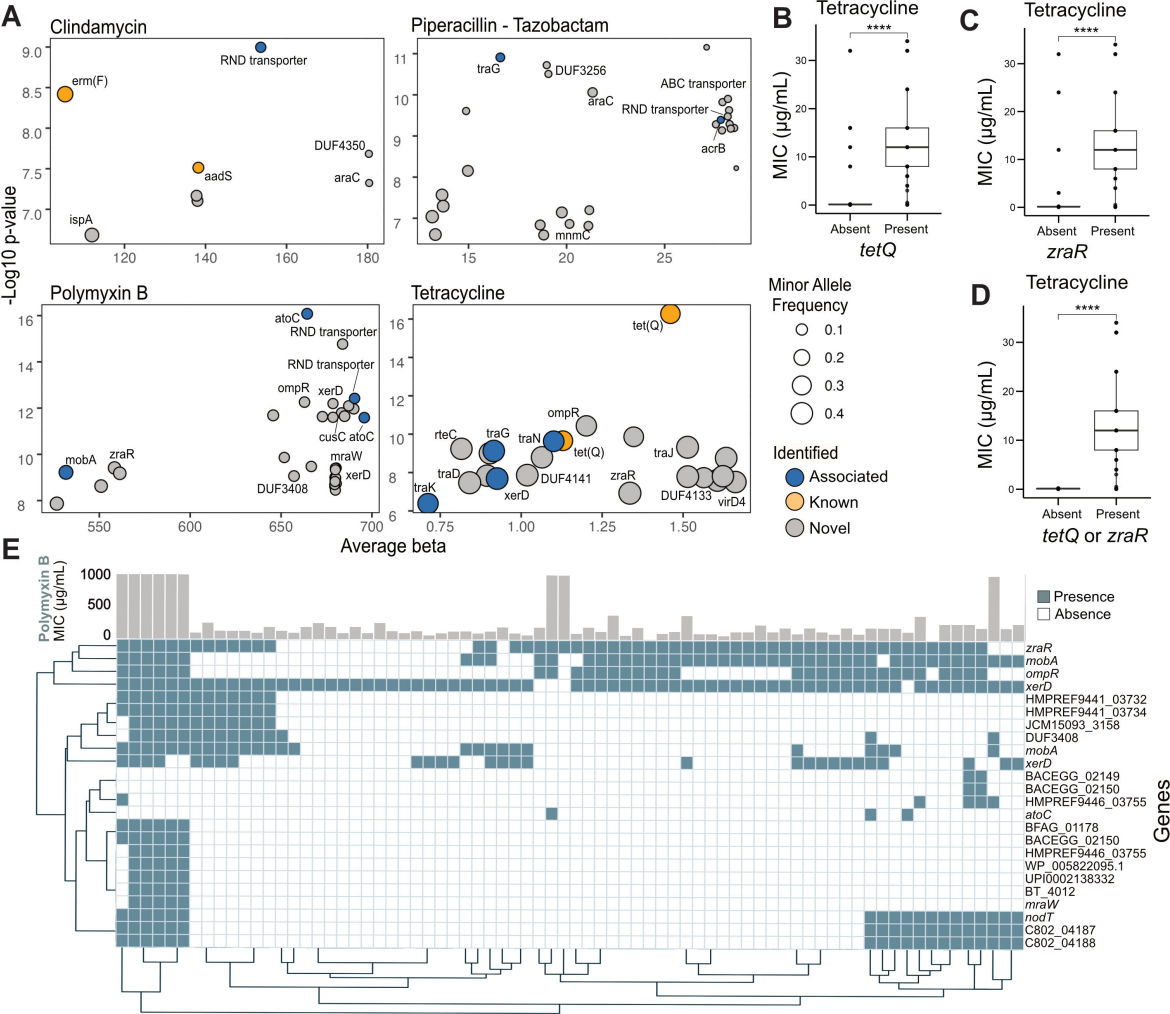
Antimicrobial resistance profiling identifies putative antimicrobial resistance genes. (**A**) Dot plot of microbial genome-wide association study (mGWAS) using unitigs of all *B. fragilis* isolates tested for antimicrobial susceptibility (*n* = 78). The Y axis shows the maximum -log_10_P value, and the X axis shows the average beta coefficient, a measure of biological effect size. Dot size indicates minor allele frequency, and color represents the number of isolates carrying the corresponding gene. Associations are shown for resistance to clindamycin, imipenem, piperacillin-tazobactam, polymyxin B, and tetracycline. (**B**) Box plots comparing MICs (μg/mL) with the presence of known antimicrobial resistance gene, *tetQ* (Welch’s t-test, *P* = 1.2e-05). (**C–D**) Box plots showing MIC comparisons for (C) *zraR* (Welch’s t-test, *P* = 3.1e-05) and the combination of (**D**) *tetQ* or *zraR* (Welch’s t-test, *P* = 1e-07, ****P < 0.0001), associated with the resistance phenotype. (**E**) Heat map of gene presence-absence per isolate for genes associated with resistance to polymyxin B, along with corresponding MIC values (μg/mL). Clustering is Euclidean and used solely for visualization purposes.

We next examined whether the presence of known AMR genes correlated with observed phenotypic resistance. Although genes such as *tet(Q), erm(F),* and *bla* were detected in many isolates, their presence did not consistently align with resistance profiles (Fig. 4B through D). This observation suggests that additional or previously uncharacterized genes may contribute to the phenotypes observed in *B. fragilis*. To explore this, we conducted a microbial pangenome-wide association study (mGWAS) using unitigs, a non-redundant representation of k-mers (21), across 78 *B. fragilis* isolates (Table S2) (22). To reinforce the validity of our approach, we successfully identified genes previously linked to AMR in *B. fragilis*, such as *ermF* and *aadS,* associated with clindamycin resistance, and *tetQ,* associated with tetracycline resistance (18, 23–25). Additionally, we identified a positive association between tetracycline resistance and the presence of the conjugative transposon, CTn341 (26). Elements associated with the transfer, regulation, mobilization, and integration of the CTn341 in the presence of tetracycline included *tra* genes (*traG, traK,* and *traJ*), *rteC*, *virD4,* and *xerD*, respectively (Fig. 4A) (27). Notably, genes associated with conjugative transposons were also identified in polymyxin B resistance (e.g., *xerD*, *traG*, *virD*, and *mobA*) (Fig. 4A). These findings emphasize that conjugative elements, widespread in *Bacteroides* spp. and other bacteria, represent a primary means for the acquisition of AMR genes from other species (28). Hence, the characterization of resistance profiles among these strains is crucial in understanding potential implications for human health (17). This is particularly concerning as extra-intestinal or *B. fragilis* strains, frequently isolated from clinical infections, increasingly pose a serious threat due to their multiple AMR mechanisms (29, 30).

### Identification of putative antimicrobial resistance genes in *B. fragilis* strains

In our mGWAS analysis, we identified additional resistance genes linked to AMR in *Bacteroides* and other species. We classified these genetic determinants as follows: “associated” for genes previously linked to AMR in non-*Bacteroides* species, “known” for genes described in the literature in *Bacteroides*, and “novel” for genetic determinants first identified in this study. We observed significant associations between polymyxin B, clindamycin, and piperacillin-tazobactam resistance and the presence of *cusC* or efflux Resistance-Nodulation-Division (RND) efflux transporters (Fig. 4A). These multi-drug efflux systems are widely distributed in bacteria (31–33) and are often regulated by *araC* transcriptional regulators (34), which we found to be associated with clindamycin and piperacillin-tazobactam resistance (Fig. 4A). Furthermore, for the antimicrobial agents tetracycline and polymyxin B, we also found an association with the previously described "envelope stress response system" gene *zraR*, which confers intrinsic antibiotic tolerance to tetracyclines and other classes of antimicrobial agents in *E. coli* (Fig. 4B through D) (35). We also found mutations in genes belonging to the outer membrane porin (*omp*) family, associated with resistance to polymyxin B and tetracycline (Fig. 4A and E). Although mutations in these proteins have been associated with increased antibiotic efflux, their primary role in AMR has been primarily attributed to regulating membrane permeability (36–38). Finally, our analysis unveiled several hypothetical genes and genes with domains of unknown function (DUF), including DUF4141, DUF4350, DUF3256, DUF4133, and DUF3408 (Fig. 4A). Further research is needed to elucidate the underlying mechanisms governing the regulation and expression of these genetic determinants in *B. fragilis* across diverse environments.

## DISCUSSION

Antimicrobial resistance in extra-intestinal *B. fragilis* strains is a major concern for clinical treatment. However, our findings show that resistance determinants are not confined to pathogenic strains. Historical trends reveal a marked increase in AMR gene prevalence since the early 2000s, consistent with the widespread antibiotic use and prior work describing the accumulation of resistance in gut-resident *Bacteroides* (14, 16). Our study extends these observations by demonstrating that intestinal and extra-intestinal isolates exhibit comparable resistance phenotypes, including similar MIC distributions and beta-lactamase activity.

This work builds on prior studies, such as those of Abigail Salyers, who characterized AMR determinants and conjugative transposons in *B. fragilis*, by directly comparing isolates from both gut and infection contexts (15–17, 19). This highlights the relevance of intestinal *B. fragilis* as a reservoir of AMR determinants that under certain conditions, such as the extra-intestinal environment, become a trait detrimental to the human host in disease states. In Gram-negative bacteria, such as *E. coli*, phylogenetically specific distributions of AMR genes segregate strains by etiology; for example, uropathogenic *E. coli* (UPEC) segregate from *E. coli* associated with bloodstream infections (39, 40). Contrary to this paradigm, we found no phylogenetic clustering by isolation site or resistance pattern in *B. fragilis*, highlighting the broad distribution of AMR determinants across the species. These results challenge the prevailing assumption that resistance primarily emerges during extra-intestinal infection, instead supporting a model in which intestinal strains act as reservoirs of resistance traits.

Using microbial genome-wide association analysis, we identified not only known resistance genes (*tetQ*, *ermF*, and *aadS*) but also novel elements, including conjugative transposons (e.g., CTn341), multidrug efflux transporters, and regulatory systems such as AraC-like transcription factors and two-component signaling modules. We also discovered mutations in outer membrane porin (Omp) family genes, associated with resistance to imipenem, polymyxin B, and tetracycline. Previous studies have shown the presence of at least 10 porin-forming proteins in *B. fragilis* strains potentially responsible for carbapenem resistance (41). The ability of *B. fragilis* to regulate porin expression under stress conditions represents an important AMR mechanism, compromising antibiotic susceptibility to first-line treatments currently used in the clinic for severe *B. fragilis* infections (e.g., imipenem). In *E. coli*, AraC functions as a transcriptional regulator involved in responses to DNA repair induced by DNA-damage agents like metronidazole and mitomycin; however, in *B. fragilis*, no prior association has been made between AraC and resistance to antimicrobial agents, such as metronidazole, which remains an effective treatment for most anaerobic infections.

Altogether, we identified several potential genes related to *B. fragilis* AMR through mGWAS analysis. Importantly, many of these genes—including hypothetical proteins and domains of unknown function (DUFs)—remain uncharacterized, emphasizing the need for future functional studies. Understanding how these loci contribute to resistance mechanisms will be essential for predicting the emergence of multidrug-resistant strains and developing effective countermeasures.

Our findings underscore the importance of considering gut commensals such as *B. fragilis* as critical reservoirs for AMR dissemination within the microbiota. The comparable resistance profiles observed between intestinal and extra-intestinal isolates highlight the potential for horizontal gene transfer events that could seed pathogenic strains with resistance determinants. These dynamics are especially concerning, given the high rates of beta-lactamase activity and the detection of conjugative elements capable of mobilizing resistance genes across species boundaries. Moreover, the identification of envelope stress response systems, multidrug efflux transporters, and porin mutations suggests that *B. fragilis* may deploy multiple, complementary strategies to withstand antimicrobial pressure.

This work expands our understanding of *B. fragilis* as both a beneficial commensal and a potential pathobiont, highlighting its capacity to transition into a clinically relevant pathogen with complex resistance profiles. Our results advocate for AMR surveillance strategies that extend beyond clinical isolates to include the gut microbiota, particularly as selective pressures from antibiotic use in the community can enrich resistance reservoirs even in asymptomatic hosts. By integrating long-term trends, phenotypic resistance data, and genomic analyses, this study provides a framework for future investigations into AMR evolution in *B. fragilis* and similar opportunistic pathogens. Functional studies of newly identified loci, including DUFs and regulatory genes, will be essential to fully understand the landscape of resistance in this key member of the microbiota. Continued genomic and phenotypic characterization of both intestinal and clinical *B. fragilis* strains will be critical to prevent the emergence of multi-drug-resistant isolates in both community and healthcare settings.

## MATERIALS AND METHODS

### Bacterial strains and culture conditions

All bacterial strains used in this study are listed in Table S3. *B. fragilis* type strain, NCTC 9343, was obtained from the American Type Culture Collection (ATCC). *B. fragilis* strains were grown anaerobically (10% H_2_, 10% CO_2_, 80% N_2_; Coy Lab Products) at 37°C in brain heart infusion (BHI) broth (BD Biosciences) supplemented with 5 µg/mL hemin (Sigma), and 0.5 µg/mL vitamin K (Sigma) (BHI-S).

### Sample collection and isolation of *B. fragilis* strains

Samples from healthy donors and patients were collected with the approval of the University of California, San Diego, Institutional Research Board and with written informed consent signed by subjects prior to sample collection. Isolates were collected under IRB #141853, #150675, and #190012, and extra-intestinal *B. fragilis* strains were collected from the Center for Advanced Laboratory Medicine (CALM) under IRB #160524. *B. fragilis* strains from healthy donors were generously provided by Dr. Eric J. Alm (Massachusetts Institute of Technology) (42). The historical *B. fragilis* strains were curated from the laboratory of Dr. Abigail Salyers (University of Illinois at Urbana-Champaign) (14, 43) and generously provided by Dr. Eric Martens (University of Michigan) (44).

Newly sequenced strains were isolated in the laboratory as previously described (5). Briefly, the strains were isolated from fecal material and homogenized in 30% glycerol/0.1% cysteine and plated in BHI-S with gentamicin (100 µg/mL). Colonies were picked and restreaked for further identification using *Bacteroides* species-specific primers by qPCR (Table S4). Further confirmation was performed by Sanger sequencing using primers for 16S rRNA, 27F, and 1492R (33). *B. fragilis* strains were banked in 50% glycerol/50% BHI-S and stored at −80°C for downstream analysis.

### Antimicrobial resistance assays by E-test

*B. fragilis* strains of interest were streaked and grown anaerobically for 48 h at 37°C on BHI-S agar plates. Antimicrobial susceptibility testing by Etest was performed per the manufacturer’s instructions (Fisher Scientific) and Clinical Laboratory Standards Institute (CLSI) guidelines (45). Briefly, a 0.5–1.0 McFarland suspension was prepared and plated onto Brucella agar with 5% Sheep Blood, Hemin, and Vitamin K (Fisher Scientific); Etest strips were applied and MICs read as specified. Antimicrobial resistance testing for the penicillin group (beta-lactamase activity) was performed using a chromogenic cephalosporin, Nitrocefin (Sigma), following the vendor’s protocol.

### Phylogenetic tree construction using a core genome alignment

To construct a phylogenetic tree from the core genome alignment, we first identified and aligned the core genes shared among the isolates. Briefly, assemblies were processed using Panaroo (version 1.2.10) under strict mode to generate a core genome alignment (core threshold ≥95%). Next, IQ-TREE (version 2.2.0.3) was used to infer a maximum-likelihood phylogeny. ModelFinder, as implemented in IQ-TREE, was employed to automatically select the best-fit substitution model. The tree was built using the selected model (e.g., GTR + F + R4), with branch support estimated by 1,000 ultrafast bootstrap replicates. The resulting tree was midpoint-rooted using ggtree (version 3.2.1) in RStudio (version 2024.04.2 + 764) for the final visualization.

### Annotation of isolates using AMRFinder

To identify antimicrobial resistance genes, each genome assembly was analyzed using AMRFinderPlus (version 3.10.18), following the recommended parameters provided by the software. Briefly, assemblies were submitted in FASTA format to AMRFinderPlus with default settings, which include a minimum sequence coverage threshold of 90% for target genes and a minimum identity of 90%. Output files were parsed to extract resistance gene annotations, which were subsequently collated into a single table for downstream comparative analyses. We examined gene presence alongside isolation dates from our *B. fragilis* strain collection to quantify resistance gene detention by decade.

### Microbial genome-wide association study

Antimicrobial resistance profiling was conducted using genomic data from 78 *B. fragilis* whole genome assemblies to identify putative antimicrobial resistance genes. The association study was conducted using FastLMM as implemented in Pyseer (version 1.3.9) (46) using a kinship matrix derived from the phylogenetic tree from the core genome alignment to account for population structure. We generated a table of all variable-size unitigs present in the isolates, generated by unitig-caller with an average unitig size of 61 bp (SD: 191 bp), and identified significant unitigs applying Holm’s correction to the unitig presence/absence p-values using the total number of unitigs as the test count. We display results at the gene level for interpretability. Because compacted unitigs can span gene boundaries or repetitive loci, multi-mapping is expected; in such cases, we retain all valid mappings. We generated a table of all unitigs present in the isolates, generated by unitig-caller, and identified significant unitigs applying Holm’s correction to the unitig presence/absence of *P*-values using the total number of unitigs as the test count. Annotations and gene identifications were performed using Bakta. For visualization and analysis of the genomic data, we generated it with ggplot2 in RStudio. Dot plots were used to depict the results of a genome-wide association study (GWAS), where unitigs from all isolates were mapped to genes associated with resistance to clindamycin, imipenem, piperacillin-tazobactam, polymyxin B, and tetracycline. Boxplots were employed to compare the minimum inhibitory concentrations (MICs) of antimicrobial agents (μg/mL) against known antimicrobial resistance genes as well as additional genes identified in this study. MIC values were determined through standard antimicrobial susceptibility testing methods. Furthermore, a heatmap was constructed to illustrate the presence or absence of genes linked to polymyxin B resistance across all isolates, accompanied by corresponding MIC values (μg/mL). Each row in the heatmap represented an isolate, with columns indicating the presence or absence of specific resistance genes.

## SUPPLEMENTAL MATERIAL

Fig. S1 iai.00529-25-s0001.docx Average number of antimicrobial resistance genes per decade among *B. fragilis* strains.

Fig. S2 iai.00529-25-s0002.docx Antimicrobial gene distribution among intestinal and extra-intestinal *B. fragilis* isolates.

Supplemental tables iai.00529-25-s0003.xlsx Tables S1 to S5.

ASM does not own the copyrights to Supplemental Material that may be linked to, or accessed through, an article. The authors have granted ASM a non-exclusive, world-wide license to publish the Supplemental Material files. Please contact the corresponding author directly for reuse.
