# Supplementary material for: Antimicrobial resistance is widespread among intestinal and extra-intestinal Bacteroides fragilis strains
Source: Infect Immun. 2025 Nov 24;93(12):e00529-25. doi: 10.1128/iai.00529-25 (PMC12707147; doi:10.1128/iai.00529-25)
Supplement: Fig. S1 — Average number of antimicrobial resistance genes per decade among B. fragilis strains. [file iai.00529-25-s0001.docx]

##


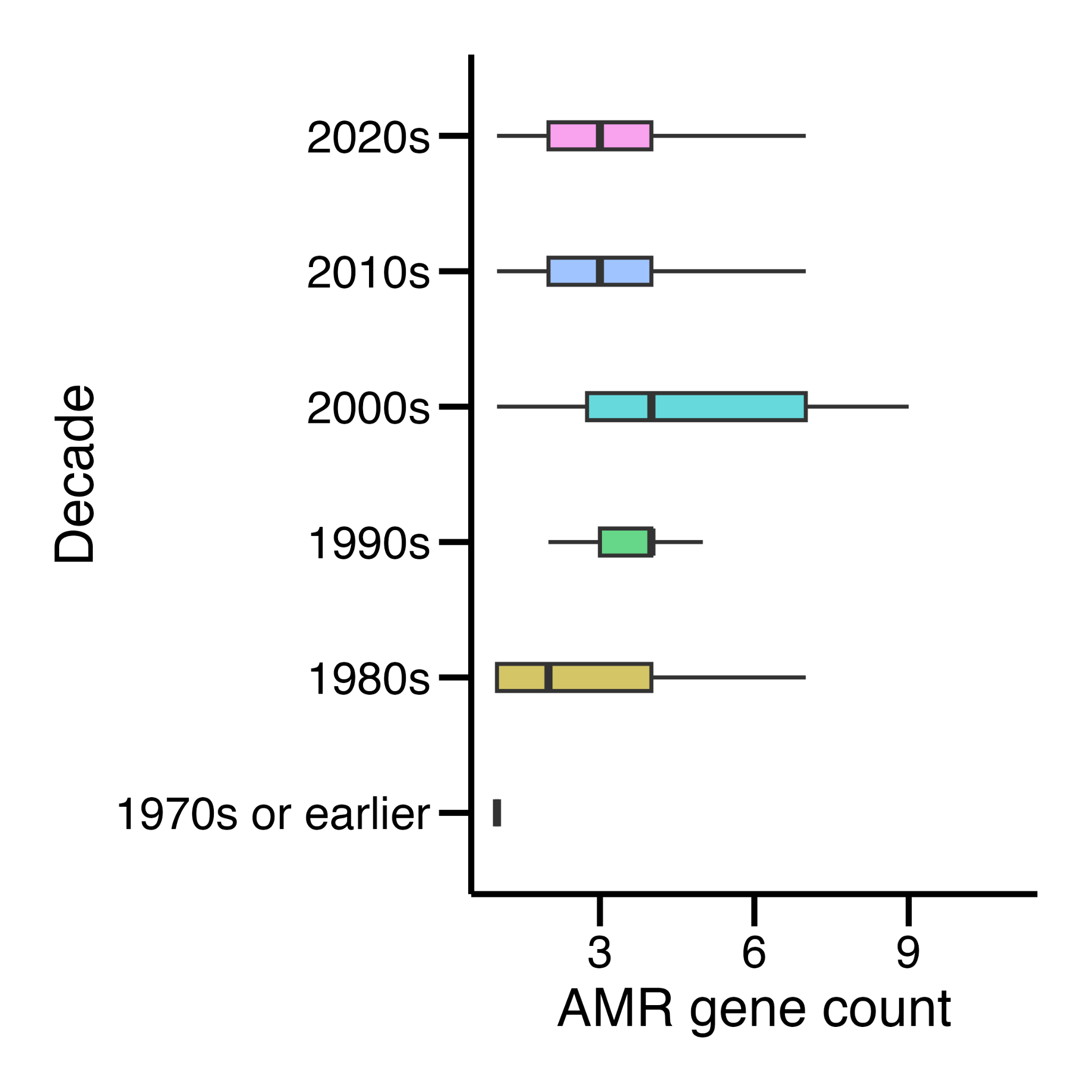


### **Supplemental Figure 1. Average number of antimicrobial resistance genes per decade among *B. fragilis* strains.** Boxplot of average number of antimicrobial resistance genes in each isolate grouped by decade of isolation (1970s or earlier = 6, 1980s = 37, 1990s = 9, 2000s = 28, 2010s = 182, 2020s = 132). Associated data can be found on **Supplemental Table 5.**
