# Supplementary material for: Antimicrobial resistance is widespread among intestinal and extra-intestinal Bacteroides fragilis strains
Source: Infect Immun. 2025 Nov 24;93(12):e00529-25. doi: 10.1128/iai.00529-25 (PMC12707147; doi:10.1128/iai.00529-25)
Supplement: Fig. S2 — Antimicrobial gene distribution among intestinal and extra-intestinal B. fragilis isolates. [file iai.00529-25-s0002.docx]

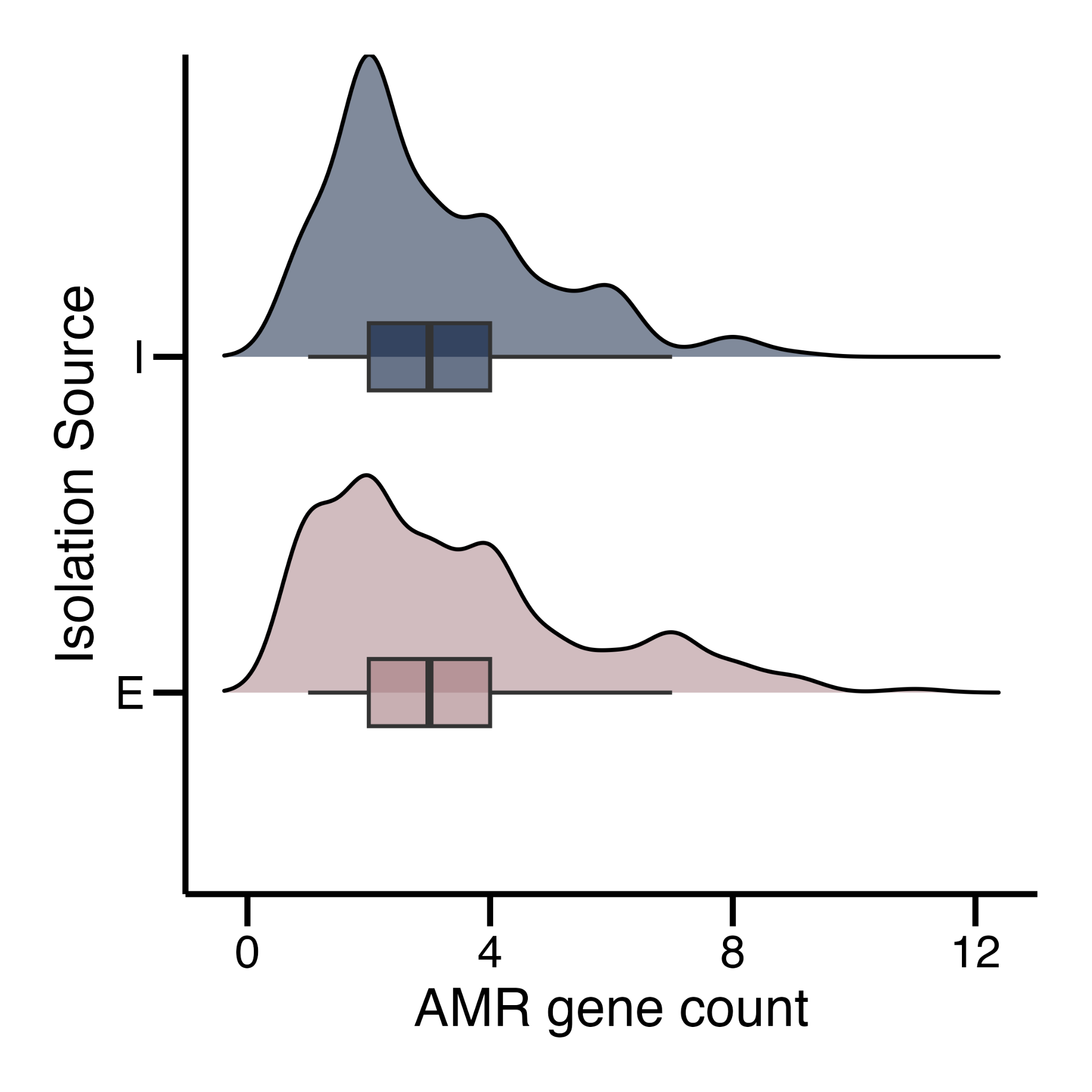


### **Supplemental Figure 2. Antimicrobial gene distribution among intestinal and extra-intestinal *B. fragilis* isolates.** Boxplot and density curve of average number of AMR genes per isolate grouped by isolation source, intestinal (I) or extra-intestinal (E).
